# Laboratory Diagnosis of Tick-Borne African Relapsing Fevers: Latest Developments

**DOI:** 10.3389/fpubh.2015.00254

**Published:** 2015-11-11

**Authors:** Aurélien Fotso Fotso, Michel Drancourt

**Affiliations:** ^1^Aix Marseille Université, URMITE, UMR 6236, CNRS 7278, IRD 198, INSERM 1095, IFR 48, Méditerranée Infection, Faculté de Médecine, Marseille, France

**Keywords:** diagnosis, laboratory, relapsing fever borreliae, Africa, point-of-care

## Abstract

In Africa, relapsing fevers caused by ectoparasite-borne *Borrelia* species are transmitted by ticks, with the exception of *Borrelia recurrentis*, which is a louse-borne spirochete. These tropical diseases are responsible for mild to deadly spirochetemia. Cultured *Borrelia crocidurae*, *Borrelia duttonii*, and *Borrelia hispanica* circulate alongside at least six species that have not yet been cultured in vectors. Direct diagnosis is hindered by the use of non-specific laboratory tools. Indeed, microscopic observation of *Borrelia* spirochaeta in smears of peripheral blood taken from febrile patients lacks sensitivity and specificity. Although best visualized using dark-field microscopy, the organisms can also be detected using Wright–Giemsa or acridine orange stains. PCR-based detection of specific sequences in total DNA extracted from a specimen can be used to discriminate different relapsing fever *Borreliae*. In our laboratory, we developed a multiplex real-time PCR assay for the specific detection of *B. duttonii/recurrentis* and *B. crocidurae*: multispacer sequence typing accurately identified cultured relapsing fever borreliae and revealed diversity among them. Other molecular typing techniques, such as multilocus sequence analysis of tick-borne relapsing fever borreliae, showed the potential risk of human infection in Africa. Recent efforts to culture and sequence relapsing fever borreliae have provided new information for reassessment of the diversity of these bacteria. Recently, matrix-assisted laser desorption/ionization time-of-flight mass spectrometry has been reported as a means of identifying cultured borreliae and of identifying both vectors and vectorized pathogens such as detecting relapsing fever borreliae directly in ticks. The lack of a rapid diagnosis test restricts the management of such diseases. We produced monoclonal antibodies against *B. crocidurae* in order to develop cheap assays for the rapid detection of relapsing fever borreliae. In this paper, we review point-of-care diagnosis and confirmatory methods.

## Introduction

In Africa, relapsing fever borreliae are neglected vector-borne pathogens responsible for various febrile presentations and are most commonly suspected in malaria-like symptoms (1). They are ectoparasite-borne infections that are transmitted by ticks, with the exception of *Borrelia recurrentis*, which is a louse-borne spirochete (1). As a result, this latter organism will not be considered in this review, which focuses on tick-borne borreliae. Relapsing fevers are a concern for public health in local populations as well as for foreigners visiting endemic African countries. Currently, three cultured tick-borne species (*Borrelia crocidurae*, *Borrelia duttonii*, and *Borrelia hispanica)* (2) are in circulation, in addition to the as yet uncultured *Borrelia merionesi* in *Ornithodoros* ticks in Morocco (3), *Candidatus Borrelia algerica* in febrile patients in Algeria (4) and an unnamed new species in *Ornithodoros porcinus* ticks in Tanzania (5), in the blood of *Spheniscus demersus* penguins in South Africa (6), in *Rhipicephalus evertsi* ticks from Nigeria (7), and new *Borrelia* species distinct from the Lyme disease and recurrent fever groups detected in *Amblyomma cohaerens* in Ethiopia (8). *B. hispanica* is prevalent in the north, *B. crocidurae* in the west, and *B. duttonii* in the east of Africa (1, 2). However, several species of *Borrelia* may circulate in the same geographic region (3). All species are transmitted by the bite of *Ornithodoros* soft ticks (9, 10). Since relapsing fever borreliae can present with fever, they are often misdiagnosed as malaria (11). Moreover, relapsing fever borreliae may form part of mixed infections, further complicating the diagnosis (12). A relapse within days is the clinical hallmark of these infections, causing mild to deadly septicemia and miscarriage in people exposed to endemic regions (1). The clinical picture initially includes a fever over 39°C with chills and polyalgia; it may also include vomiting, abdominal pain, and diarrhea. Physical examination may find rash, splenomegaly, and hepatomegaly (2). All species may cause iritis, iridocyclitis, uveitis, and central nervous system infection. The mortality rate is estimated to be between 2 and 5%, depending on the causative *Borrelia* species, the highest mortality rate being observed with *B. recurrentis* (1). The most recent epidemiological data indicate that 43.92 million people living in rural Africa in endemic countries and 19.17 million travelers are at risk of relapsing fever in west and north African countries (3). The incidence of tick-borne relapsing fever has been measured at 11% in rural Senegal (9). Due to its sudden onset, and because the first fever attack is the most dangerous, lengthy diagnostic procedures, including *in vitro* culturing and animal inoculation, cannot be considered for routine diagnosis. Currently, detection of relapsing fever borreliae in Africa relies upon the observation of spirochaeta in smears of peripheral blood; however, the high morphological similarity between species does not allow for identification at the species level. Molecular methods, detecting single nucleotide polymorphisms in the 16S rRNA and *flaB* genes, 16S-23S ribosomal RNA intergenic spacer (IGS), multispacer sequence typing (MST), multilocus sequence typing (MLST), and multiplex quantitative real-time PCR (4, 13, 14), may not be routinely available in most endemic regions (15). Nevertheless, rapid diagnosis of relapsing fever is warranted, since these cases require specific treatment and prophylaxis in order to avoid contact with small rodents and their ticks (12). Relapsing fevers remain undiagnosed partly due to the lack of point-of-care (POS) diagnostic tools in endemic countries (15), resulting from the fastidious nature of the tools and the lack of attention from doctors and microbiologists toward febrile patients returning from endemic areas. Here, we review the tools that are currently available for the diagnosis of relapsing fever borreliae in hosts in Africa.

## Rapid Diagnosis at the Point-of-Care

**Figure 1:**
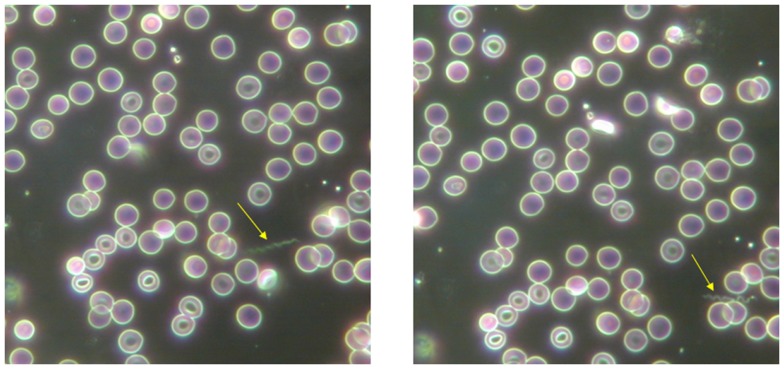
**Presence of extraerythrocytic *Borrelia crocidurae* (arrow) in a blood smear from guinea pigs using dark-field microscopy**. Magnification ×400.

**Figure 2:**
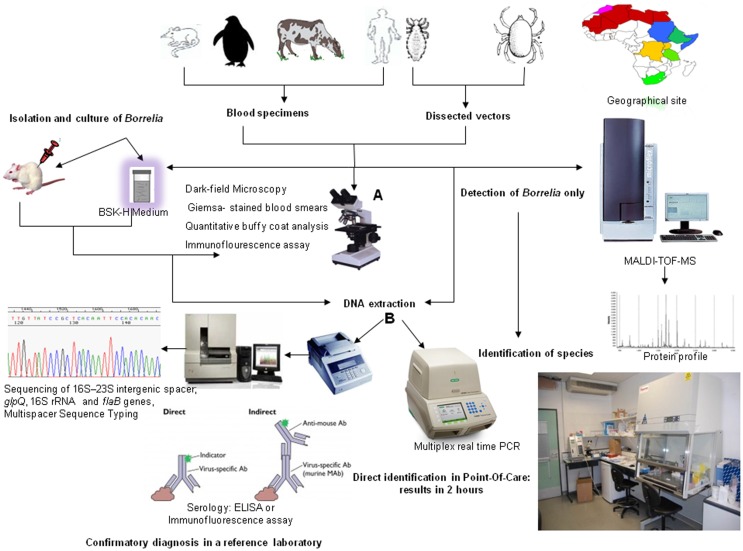
**Diagnosis of African relapsing fever: a review**. Conventional microscopic identification **(A)** and molecular identification **(B)**.

The gold-standard diagnosis for relapsing fever borreliae is direct microscopic visualization of borreliae in a Giemsa-stained thick blood smears (12, 16). Borreliae are best detected in blood obtained while a patient is febrile. During subsequent febrile episodes, the number of circulating spirochaeta decreases, making it harder to detect them on a peripheral blood smear (16). One study, using thick blood smears from febrile cases stained with Giemsa and observed in 200 oil immersion fields (×1000) (equivalent to about 0.5 μL blood), determined that during the febrile episode, the blood-borne inoculum was 10^3^–10^5^ borreliae per mL (9, 16). This figure indicates that conventional microscopic examination of a blood drop yields only one *Borrelia* every 10 microscopic fields. Accordingly, microscopic examination of red blood cells for *Plasmodium* may easily overlook borreliae, which are free in the plasma (17) or sticking to blood cells (Figure 1). Direct detection either of motile spirochaeta by dark-field microscopy or staining of borreliae by fluorescent antibody methods in such tissues may be used (18, 19). The inability of dark-field microscopic analysis to detect spirochaeta in 100 field-collected ticks (16 infected ticks) compared with PCR (22 infected ticks) (20) illustrated the need for more sensitive tests. To provide simple, fast, cheap, and sensitive diagnoses using equipment that is available in small health centers, a method based on enrichment of bacteria by centrifugation and detection by Giemsa staining was developed, which is capable of detecting fewer than 10 spirochaeta per mL of blood (12, 21). PCR-based detection of specific sequences is the modern method for POC laboratory diagnosis (15). Total DNA is most often extracted from blood using a QIAamp DNA Micro kit according to the manufacturer’s protocol (QIAGEN, Hilden, Germany) (4, 22). Because of the close genetic and genomic proximity of the relapsing fever borreliae, as illustrated by 16S rRNA gene sequence variability ≤1%, confirmed by *B. duttonii* and *B. recurrentis* genomics, indicating that the two organisms belonged to the same bacterial species (22–24), molecular tests may detect relapsing fever borreliae without providing species identification. Observations that the glycerophosphodiester phosphodiesterase (*glp*Q) gene and its antigenic protein product were present in relapsing fever borreliae and absent in Lyme disease borreliae led to the development of group-specific molecular and serological tests (25). Detection of *Borrelia* flagellin DNA (*flaB*) by real-time PCR amplification from the blood is highly sensitive and specific, detecting as few as 10 borreliae per mL of blood (26). Real-time PCR can be efficiently used in POC settings, as shown in rural Senegal (15, 27). In this example, extracted blood DNA could be stored at 4°C until used for PCR amplification. Ready-to-use lyophilized reagents can be prepared in a core-laboratory and shipped to rural POC facilities (15). The lyophilized mixtures can be stored for at least 2 months at 20°C without loss of activity (Figure 2). The same approach has recently been used for the POC molecular diagnosis of Buruli ulcers in order to facilitate the diagnosis of *Mycobacterium ulcerans* infection in endemic countries (27). While commercially available real-time PCR solutions may be beyond the financial capacities of some countries, specific financing programs are available to promote advanced molecular diagnosis in endemic countries, as illustrated by rural POC facilities (15).

## Confirmatory Diagnosis in the Reference Laboratory

### Molecular Typing

Molecular typing confirms *Borrelia* and allows for fine species identification. Several approaches, including analysis of the IGS located between the 16S rRNA and 23S rRNA genes, only explored the variability between *B. duttonii* and *B. recurrentis* (28). Moreover, IGS sequences overlapped between one *B. duttonii* phylogenetic group and one *B. recurrentis* group, with a second overlap being revealed upon subsequent analysis of further material (28). In Mali, the inclusion of MLST revealed the endemic foci of tick-borne relapsing fever borreliae and the potential risk for human infection (14). *Borrelia* contains numerous linear and circular plasmids (23, 29, 30). MLST looks for differences between plasmid profiles and DNA sequence data. MST looks for differences between five IGSs in *B. crocidurae*, *B. duttonii*, and *B. recurrentis* genomes. It is a suitable PCR-sequencing-based method for identifying and genotyping relapsing fever borreliae in Africa in both vectors and clinical specimens (13). *B. hispanica* is responsible for relapsing fever borreliae in Spain (31) and previous molecular studies have identified this species in patients with unexplained fever in north-western Morocco (32). However, local transmission has not been detected in Morocco since 1976 (33), with the exception of one traveler returning from Spain and Morocco with a *B. hispanica* infection (34). Although not present in Europe or near its boundaries, other relapsing fever borreliae species are of interest to European physicians because of the increase in international travel and cases of European tourists importing relapsing fevers. *B. crocidurae* has been detected in travelers returning from Senegal (35, 36) and Mali (37), and other relapsing fever borreliae have been detected in travelers returning to Europe from Africa over the past 15 years (Table 1). Relapsing fever borreliae are therefore considered as emerging diseases and should consequently be considered in all febrile patients returning from endemic countries.

**Table 1:** **Diagnosis of relapsing fever borreliae in travelers returning to Europe from Africa over the past 15 years**.

Reference laboratory	Countries of exposure	Methods	Reference
Infectious Diseases and Tropical Medicine ward in North Hospital, Marseille, France	Senegal	Microscopic Molecular tests	(35)
The Emergency Department of American Hospital of Paris, France	Mali	Microscopic	(37)
Clinic and Laboratory of Infectious Diseases, University of Siena, Siena, Italy	Senegal	Microscopic Molecular tests	(36)
Biology laboratory of the Centre Hospitalier d’Argenteuil, France	Senegal	Microscopic Molecular tests	(38)
Service de Médecine Interne, Groupe Hospitalier Mutualiste de Grenoble, France		Lyme disease serodiagnostic assays (ELISA and Western blot) Culture	
University Hospital of Antwerp, Belgium	Senegal	Molecular tests	(39)
Université de la Méditerranée, Marseille, France	
Hôtel-Dieu Hospital, Paris, France	Morocco	Microscopic	(34)
Hôpital de Mantes in Mantes-la-Jolie, France	Mali	Molecular tests	
Avicenne Hospital in Bobigny, France	Mauritania	
Service de maladies infectieuses et réanimation médicale, CHU Pontchaillou, Rennes, France	Senegal	Microscopic Molecular tests	(40)
Infectious Diseases and Tropical Medicine ward in North Hospital, Marseille, France	Ethiopia	Molecular tests Immunofluorescence assay Lyme disease serodiagnostic assays (ELISA)	(41)

### Techniques for Epidemiological Study

Molecular typing is used for species identification of *Borrelia* in epidemiological studies (13, 14, 28). In Ethiopia, molecular investigation of 284 cattle ticks found a potential new *Borrelia* species distinct from both the relapsing fever group and Lyme borreliae (8). In Ethiopia, *B. recurrentis* DNA has also been found in 23% of head lice from patients with louse-borne relapsing fever (42).

### Xenodiagnosis

Because relapsing fever borreliae are vector-borne pathogens, it is possible to use xenodiagnosis to detect the causative *Borrelia* in the vector. We recently developed a protocol for the rapid detection of *B. crocidurae* in *Ornithodoros* soft ticks using matrix-assisted laser desorption/ionization time-of-flight mass spectrometry (MALDI-TOF-MS) (43). MALDI-TOF-MS is, thus, emerging as a potential tool for the rapid identification of vectors (44) and spirochaeta such as *Leptospira* (45) and *Borrelia* (46). We first extended it to the dual identification of vectors and vectorized relapsing fever borreliae directly in ticks (43). For each *Borrelia* species, a consensus pattern referred to as the mean spectrum projection (MSP) was obtained using the Biotyper MSP Creation Standard Method (Bruker Daltonics). A *Borrelia* MALDI-TOF-MS database was created for relapsing fever borreliae and yielded a unique protein profile for each species.

After the database had been developed, MALDI-TOF-MS was able to be used to identify tick species and the presence of relapsing fever borreliae in a single assay. The Borreliae database, along with a custom software program that subtracts the uninfected *Ornithodoros sonrai* profile, was used to detect *B. crocidurae*. The legs were homogenized and the supernatant was spotted onto a steel target plate in quadruplicate. Using in-house subtraction software, the MSP pattern of non-infected *O. sonrai* was removed from the pattern of infected ticks. This software normalizes the spectra and compares common peaks in infected and uninfected ticks, subsequently generating the MSP spectra before performing the subtraction. After subtraction, the list of remaining differential masses (*m/z*) was compared with the *B. crocidurae* MSP.

### Isolation and Culture

Screening tests currently consist of microscopic examination and culture of midgut tissues dissected from live vectors of relapsing fever borreliae (18). Although somewhat difficult and time-consuming, this culture is definitive for the diagnosis of spirochaetal infections in ticks. It also provides a source of new *Borrelia* strains (47). Borreliae grow at 32°C in Barbour-Stoenner-Kelly-H (BSK-H) medium supplemented with 10% heat-inactivated rabbit serum. Dark-field microscopic observation ensures the absence of any contaminant organisms and gages the growth of the borreliae. Fresh blood samples from infected patients can be cultured using BSK-H medium or by intraperitoneal inoculation of 6- to 8-week-old female laboratory BALB/c mice. Borreliae in mice are detected after 5–6 days by microscopic examination of Giemsa-stained peripheral blood smears, followed by qPCR of blood samples. Outside of Africa, it has been demonstrated that *Borrelia hermsii* (new world relapsing fever borreliae) infection in mice can be quantified by qPCR and that this technique matched the results obtained by microscopic examination of blood smears (48). In a study based on 100 field-collected *Ixodes ricinus* ticks, dark-field microscopy, culture, and PCR were shown to be comparable as procedures for detecting Lyme borreliosis spirochaeta in ticks. Thirteen ticks were found to be positive through culture in BSK-H medium, 16 ticks were found to be infected with spirochetes by dark-field microscopy, and 22 ticks were found to contain *Borrelia burgdorferi*-specific DNA by PCR using a primer set based on sequences of the flagellin gene of *B. burgdorferi* (20). This study showed the inability of culture to detect spirochaeta compared to dark-field microscopic analysis and PCR. For the culture of tick-borne relapsing fever borreliae in Africa, *B. hispanica* remained uncultured in axenic medium until 1976 (49) and *B. duttonii* until 1999 (50). *B. crocidurae*, first described in musk shrew blood in Senegal in 1917 (51), was only cultured in axenic medium in 1999 (52).

### Serology

No reliable specific serology exists for *B. crocidurae* infection. Indeed, the detection of *B. burgdorferi* and *B. recurrentis* by IgM enzyme-linked immunosorbent assay (ELISA) was used as a surrogate for *B. crocidurae* serology since it is thought that an appreciable cross-reacting antigen homology exists between these species and *B. crocidurae* (35). The *glp*Q gene has been proposed as a more specific recombinant antigen for serology, distinguishing relapsing fever borreliae from Lyme disease, but this test is not species-specific, it cannot date the infection and is not commercially available (25). It also failed to detect IgG response during early infection in some patients infected with *B. recurrentis* (25, 53). Serology is of little use for diagnosis as specific antibodies are detectable only after the first week of infection (53). Moreover, the majority of antibodies are directed toward variable membrane proteins (Vmps), subdivided into variable small proteins (Vsps) of 20 kDa and variable large proteins (Vlps) of 37 kDa in the genus *Borrelia* (54). One disadvantage of serology in endemic areas is antibodies that remain from earlier cleared infections may result in false-positive results (53). Moreover, several relapsing fever proteins are cross-reactive to *Treponema* spirochaeta and Lyme group borreliae (25). No antibody has been specifically developed for the diagnosis of African relapsing fever borreliae. Recently, we produced monoclonal antibodies (MAbs) against *B. crocidurae* and characterized two of them with a higher titer in order to develop cheap assays for the rapid detection of relapsing fever borreliae (19). By combining both MAbs into immunofluorescence assays, relapsing fever *Borrelia* and specifically *B. crocidurae* were detected in ticks and blood. These antibodies could be incorporated into a dot-blot, a cheap and stable format which is well-suited to the rapid diagnosis of relapsing fevers at the POC (15).

## Conclusion

Relapsing fevers are common diseases in local and traveling populations exposed to endemic countries in Africa. Nevertheless, they remain largely undiagnosed due to a lack of routine laboratory tests and clinical awareness. These fevers can be easily detected at POC using home-made, specific real-time PCR assays (15, 27). Fine identification can be then confirmed in a reference laboratory. Relapsing borrelioses should be incorporated to all tropical fever syndromic laboratory kits and an ethylenediaminetetraacetic acid (EDTA) blood tube, along with any ectoparasites that have been removed, should be collected for diagnosis purposes.

## Conflict of Interest Statement

The antibodies reported herein have been patented under N° FR 2015/1461399 by Aurélien Fotso Fotso, Didier Raoult, and Michel Drancourt.
